# Physical basis for the determination of lumen shape in a simple epithelium

**DOI:** 10.1038/s41467-021-25050-3

**Published:** 2021-09-23

**Authors:** Claudia G. Vasquez, Vipul T. Vachharajani, Carlos Garzon-Coral, Alexander R. Dunn

**Affiliations:** 1 Department of Chemical Engineering, Stanford, USA; 2 Biophysics Program, Stanford University, Stanford, CA USA; 3 Stanford Cardiovascular Institute, Stanford University School of Medicine, Stanford, CA USA

**Keywords:** Biophysics, Cell biology, Adherens junctions, Mechanotransduction, Tight junctions

## Abstract

The formation of a hollow lumen in a formerly solid mass of cells is a key developmental process whose dysregulation leads to diseases of the kidney and other organs. Hydrostatic pressure has been proposed to drive lumen expansion, a view that is supported by experiments in the mouse blastocyst. However, lumens formed in other tissues adopt irregular shapes with cell apical faces that are bowed inward, suggesting that pressure may not be the dominant contributor to lumen shape in all cases. Here we use live-cell imaging to study the physical mechanism of lumen formation in Madin-Darby Canine Kidney cell spheroids, a canonical cell-culture model for lumenogenesis. We find that in this system, lumen shape reflects basic geometrical considerations tied to the establishment of apico-basal polarity. A physical model incorporating both cell geometry and intraluminal pressure can account for our observations as well as cases in which pressure plays a dominant role.

## Abstract (web summary)

The formation of a hollow lumen surrounded by cells is a key developmental process that sets the shape of tissues and organs. Here, the authors show how the combined influence of geometric constraints imposed by cell packing and osmotic pressure can generate the diverse range in lumen shapes observed in different tissues.

## Introduction

Lumens, or hollow openings surrounded by sheets of cells, are a ubiquitous structural feature of metazoans. While the molecular components required for lumen formation have been characterized in detail, the physical mechanisms that underlie the initial steps in lumen formation remain less explored^1–9^. Previous work in Madin-Darby Canine Kidney (MDCK) spheroids and other systems has assumed that lumen growth occurs due to intraluminal hydrostatic pressure^10–13^. In such a scenario, lumen shape and size are governed by the Young-Laplace equation, which states that the pressure difference (*P*) between the cells and the lumen is counterbalanced by the surface tension (*ɣ*) of the lumen surface (apical faces of the cells) and inversely proportional to the lumen radius (*r*):

1 $$\triangle P=\frac{\gamma }{r.}$$

The presence of a luminal pressure is motivated by work that showed ion channels are critical for lumen formation and expansion in vitro and in vivo^14,15^, and by pressure-driven fluctuations in lumen size in some model systems^10,13^.

Importantly, a positive luminal pressure should produce convex lumen surfaces that bow outwards toward the surrounding cells. In model systems such as the developing mouse blastocyst and the bile canaliculus this convex surface curvature is well-documented^11,16^. However, published images of some model systems, for example MDCK cell spheroids and various in vivo examples of lumens such as liver bile ducts, blood vessels, and pro-amniotic cavities, demonstrate areas of concave lumen curvature, where cell apical faces are bowed into the lumen^1–3,5,17–22^. These observations suggest that a positive pressure gradient may not be the dominant contributor to the growth of all lumens.

In this study, we sought to understand the physical forces maintaining lumen shape in the context of *de novo* lumen formation. We examined the mechanics of lumen formation and expansion in MDCK cell spheroids, an archetypal cell culture model for studying lumenogenesis. Our experiments revealed that neither lumen pressure nor the actomyosin cytoskeleton were required to maintain the stability of lumen shape. Instead, we find that, in our model system, lumen shape is determined primarily by geometrical constraints arising from the creation of distinct, lumen-facing apical domains. Motivated by these observations, we developed a biophysical model in which lumen shape is determined by the combined influence of intraluminal pressure and basic geometric considerations. Our results support a unifying physical mechanism for the formation of luminal openings in a variety of physiological contexts.

## Results

### Lumens in small-size and intermediate-size MDCK spheroids are irregularly shaped

**Fig. 1:**
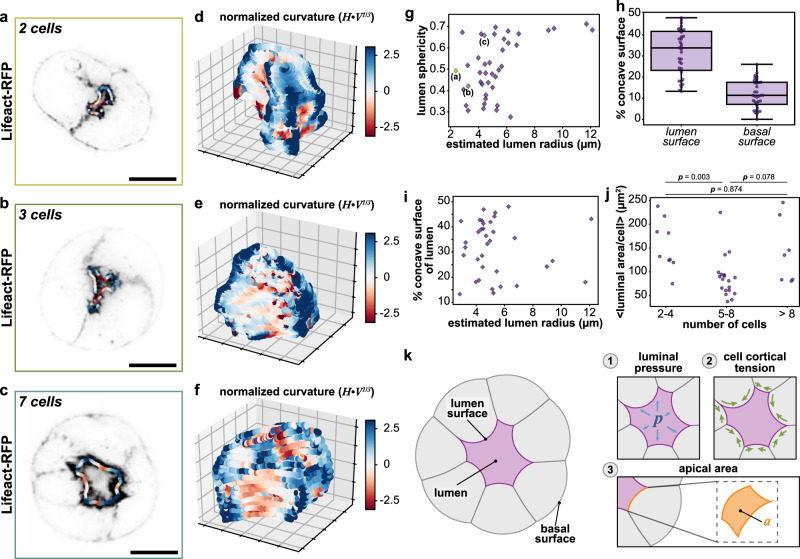
Quantification of lumen morphology. **a**–**c** Representative single-plane images of MDCK spheroids expressing Lifeact-RFP (gray). The mean lumen curvature is superimposed as a red-blue outline, where red is concave (negative local curvature) and blue is convex (positive local curvature). **d**–**f** 3D contour plots of corresponding lumen surfaces showing volume normalized (*V*) local mean curvatures (*H*), where red is concave (negative local curvature) and blue is convex (positive local curvature). **g** Lumen sphericities plotted as a function of estimated lumen radius. Estimated lumen radius (*r*) was calculated using lumen volume (*V*): *r* = (3 *V*/4*π*)^1/3^. Values for representative spheroids from **a**–**c** as indicated. **h** Percent of lumen surface (left) and basal surface (right) that is concave (negative curvature). **i** Percent of lumen surface that is concave (negative curvature) as a function of estimated lumen radius, as determined by lumen volume. **j** Mean luminal surface area per cell plotted as a function of number of cells in the spheroid (*p*-values from two-sided rank sum test). **k** Schematic depicting physical forces that could determine luminal shape. (1) luminal pressure (*p*) (2) cell cortical tension and (3) apical area (*a*). Scale bars are 10 μm. Box plot in **h** shows median, quartiles of dataset, and whiskers extending to maximum and minimum of distributions. For plots **g**–**j**, *n* = 35 spheroids. Source data are provided in the Source Data file.

We used MDCK cells as our model system to study the mechanisms that dictate lumen shape due to their ability to reliably establish apico-basal polarity, and form lumens in 3D culture in a manner that recapitulates lumenogenesis in in vivo model systems^1,5,9^. We seeded MDCK cells expressing a fluorescent marker for actin filaments (Lifeact-RFP) in the recombinant extracellular matrix Matrigel. Under these culture conditions, MDCK cells spontaneously form hollow spheroids within 24 h. To obtain high-resolution images of nascent lumens, we imaged young (18–24 h) spheroids using lattice light sheet microscopy (LLSM). This acquisition method produced images in which the two opposing apical cortices of the lumen are clearly distinguishable and separated by ~200–300 nm (Fig. 1a, b and Supplementary Video 1). We infer that cellular apical surfaces are intrinsically non-adherent, as even small fluctuations in cell shape would allow apposing apical surfaces to contact and potentially adhere. This non-stick behavior may reflect the enrichment of negatively charged sialoglycoproteins such as Podocalyxin and/or the active exclusion of cell adhesion proteins (e.g., E-cadherin)^3,9,21,23^.

To derive insight into the physical mechanisms that dictate the shape of small and intermediate-sized lumens, we used confocal microscopy to quantify the shapes of the luminal (apical) and outer (basal) surfaces of intermediate-size MDCK spheroids grown for 2 days in 3D conditions. As with two-cell to three-cell spheroids, the lumens of intermediate-sized spheroids (7–30 cells) were irregular in shape (Fig. 1c and Supplementary Fig. 1a). This observation is consistent with published shapes of MDCK lumens^1–3,5^ and other lumens observed in intact organisms^17–21^ though the irregularity of shape was not previously commented upon or explored in detail.

To determine how similar or dissimilar lumen shapes were to spheres, we calculated the sphericity *Ψ*, given by:

2 $$\psi =\frac{\pi^{1/3}(6V)^{2/3}}{A}$$

for lumen volume *V* and surface area *A*. This metric ranges from 0 (far from spherical) to 1 (exactly a sphere). The sphericity of lumens ranged from *Ψ* ~ 0.70 to very far from spherical (*Ψ* ~ 0.3) (Fig. 1g), values substantially less spherical than, for example, a cube (*Ψ* = 0.81). Further, we observed a lumen size-dependent crossover from irregular morphology to more spherical morphology: the lumens of large MDCK spheroids, with an estimated radius (calculated from lumen volume) of ~10 µm and tens of cells, abruptly transitioned to more spherical shapes with *Ψ* ~ 0.7, in agreement with published images of large lumens^6,7,9,12^.

In addition to lumen sphericity, we computed the mean curvature (*H*) at each voxel of lumen surface, normalized by lumen volume. These measurements demonstrate that lumens, even those with sphericity greater than 0.65, have areas of negative mean curvature, where the negative value denotes concave (inward) bending (red; Fig. 1d–f and Supplementary Fig. 1b). The percent of the total lumen surface area with negative mean curvature varied from 10% to over 50% (Fig. 1h). Unlike the trend for sphericity, there were no clear size-dependent trends towards a lower fraction of concavity as lumens grew larger (Fig. 1i). In contrast to the variability of lumen shape observed, the sphericity and percent concavity of the basal surfaces of the spheroids were close to 1 and 0%, respectively (Fig. 1h and Supplementary Fig. 1c).

These data are inconsistent with a model of positive luminal pressure as the sole driving mechanism to maintain the shape of small-sized and intermediate-sized lumens. However, we noted as well that the average luminal surface area per cell varied only modestly between spheroids of 2 to >8 cells, with no clear trend in comparing lumens of 2–4, 5–8, and >8 cells (Fig. 1j). This observation suggested an alternate scenario in which the apical surface area per cell was actively regulated, with apical surface area added faster than the equivalent amounts of luminal volume, thus leading to the observed irregular lumen shapes. Motivated by this possibility, we decided to more closely investigate three physical factors that could influence lumen shape: (1) intraluminal pressure, (2) cell cortical tension, and (3) a preferred apical domain size (Fig. 1k).

### Intraluminal pressure does not significantly define lumen shape stability

**Fig. 2:**
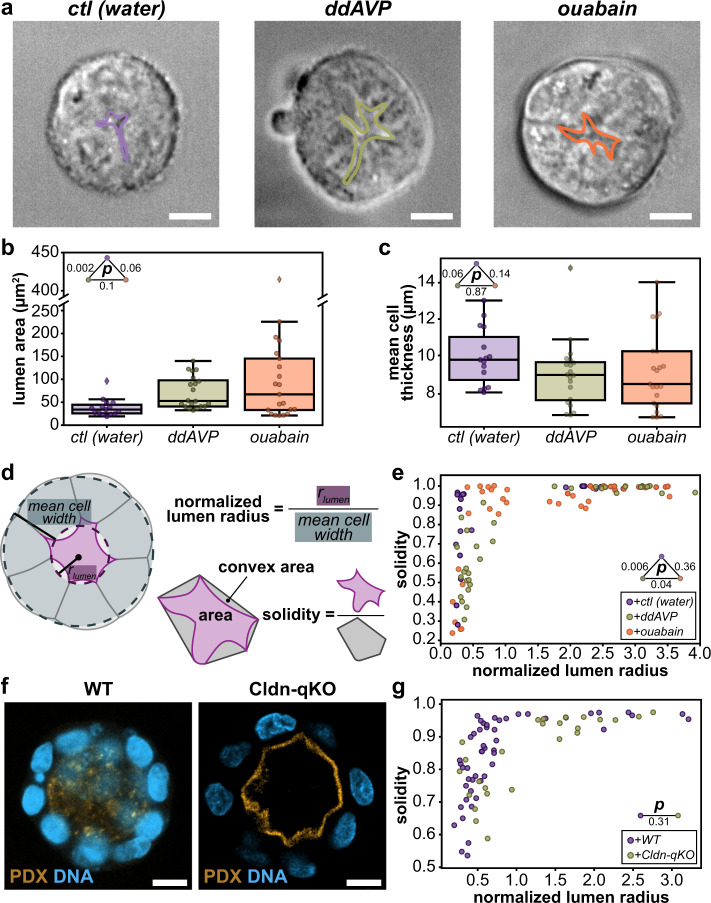
Manipulation of intraluminal pressure does not significantly affect lumen shape. **a** Representative cross-sections of MDCK spheroids grown for 3 days imaged using differential interference contrast treated with vehicle (left), ddAVP (center), or ouabain (right) for 4 h. Lumens are outlined in purple, green, and orange, respectively. **b** Quantification of cross-sectional luminal area of MDCK spheroids grown for 3 days in control, ddAVP, and ouabain conditions (*p*-values are from two-sided rank-sum test). **c** Quantification of mean cross-sectional cell thickness of MDCK spheroids grown for 3 days in control, ddAVP, and ouabain conditions (*p*-values are from two-sided rank-sum test). **d** Schematic describing solidity, a metric that reflects surface irregularity, and normalized lumen radius metric. **e** Solidity of lumens from MDCK spheroids grown for 3 days and 7 days, treated as indicated with vehicle (purple), ddAVP (green), or ouabain (orange) for 4 h as a function normalized lumen radius (ratio of estimated lumen radius determined by lumen volume and mean cell width) (*p*-values from two-sided 2D Kolmogorov–Smirnov test). **f** Representative images of fixed wildtype (WT, left) and Claudin-quintuple KO (Cldn-qKO, right) MDCK spheroids with lumens immunostained for the apical surface protein podocalyxin (PDX, orange) and DNA (blue)^26^. **g** Lumen solidity plotted as a function of normalized lumen radius for wildtype lumens (WT, purple), Cldn-qKO lumens (green) (*p*-value from two-sided 2D Kolmogorov–Smirnov test). Scale bars are 10 μm. Box plots in **b** and **c** show median, quartiles of dataset, and whiskers extending to maximum and minimum of distributions, excluding outliers (indicated with diamonds). For plots **b** and **c**, *n* = 15, 21, 20 spheroids for control, ddAVP, and ouabain conditions, respectively. For plot **e**, *n* = 48, 33, 42 spheroids for control, ddAVP, and ouabain conditions, respectively. For plot **g**, *n* = 51, 25 spheroids for WT and Cldn-qKO conditions, respectively. Source data are provided in the Source Data file.

To test how modulating intraluminal pressure affected lumen shape, we treated MDCK spheroids grown in Matrigel for either 3 or 7 days with small molecules that act to promote or inhibit apical fluid secretion: the V2 receptor agonist 1-desamino-8-D-AVP (ddAVP, 10 µM) and the Na^+^/K^+^ ATPase inhibitor ouabain (330 µM), respectively^10,24,25^ (Fig. 2a). Treatment with ddAVP for 4 h caused lumen cross-sectional area to increase compared to vehicle controls while having little change in cell thickness, consistent with the expected increase in lumen volume (Fig. 2b, c and Supplementary Fig. 2a, b). The lumens of spheroids treated with ouabain did not show a statistically significant increase or decrease in cross-sectional area, but did exhibit increased variability (Fig. 2b*, p* = 0.009) One possible explanation for this latter observation is that decreased luminal pressure due to ouabain treatment may allow lumens to more freely fluctuate in shape, thus increasing the variability of the cross-sectional area.

To further explore how these treatments altered lumen shape, we calculated the solidity of lumen cross-sectional shapes. The solidity metric is defined as the ratio of the lumen area and the area of its convex hull (Fig. 2d). A value of 1 describes completely convex shapes, such as an ellipse or a regular polygon, while lower values reflect varying degrees of surface concavity. We chose to evaluate lumen shape by this metric because it could capture the variations in lumen shape concavity that other 2D metrics we tested did not. We also calculated how similar or dissimilar individual luminal cross-sections were to circles by calculating the isoperimetric quotient (IPQ), given by:

3 $${IPQ}=\frac{4\pi a}{{p}^{2}},$$

for lumen cross-sectional area *a*, and lumen cross-sectional perimeter *p*. A value of 1 for this metric represents a perfect circle. To determine how these metrics change with lumen size we plotted them against a normalized lumen radius, the ratio between the estimated lumen radius and the mean cell width (Fig. 2d). This normalization accounts for the difference between lumens of the same size but surrounded by thicker or thinner cells. Independent of treatment with ddAVP, ouabain, or water vehicle control, the solidity and IPQ of lumens showed the same abrupt transition to values close to 1 with increasing relative lumen size (Fig. 2e and Supplementary Fig. 2c). Although control and ddAVP treated lumens were statistically distinguishable by a 2D Kolmogorov–Smirnov test, differences in lumen shape were modest in practice (Fig. 2a, e). Thus, for lumens of approximately four to tens of cells, modulating fluid pumping via ddAVP or ouabain did not strongly influence lumen shape.

To further probe this observation, we measured the solidity of lumens formed by spheroids grown from an MDCK cell line lacking the five most highly expressed claudins (quintuple knockout; Cldn-qKO)^26^. Claudins are essential components of the tight junction. A previous study showed that monolayers formed by Cldn-qKO cells showed dramatically increased small-molecule permeability relative to wildtype cells. It is therefore unlikely that Cldn-qKO cells could maintain a large pressure difference between the lumen and the exterior environment. Despite this, Cldn-qKO cells formed lumens whose solidity did not differ from wildtype to a significant degree (Fig. 2f, g and Supplementary Fig. 2e). Remarkably, lumens formed by Cldn-qKO cells were comparable or larger than those formed by wildtype (Supplementary Fig. 2f), when the opposite might be expected if pressure was important in determining lumen size and shape. We conclude that, at least in this model system, pressure alone is not the primary driver for determining lumen shape and size.

### Cell cortical tension subtly influences lumen volume but not shape stability

**Fig. 3:**
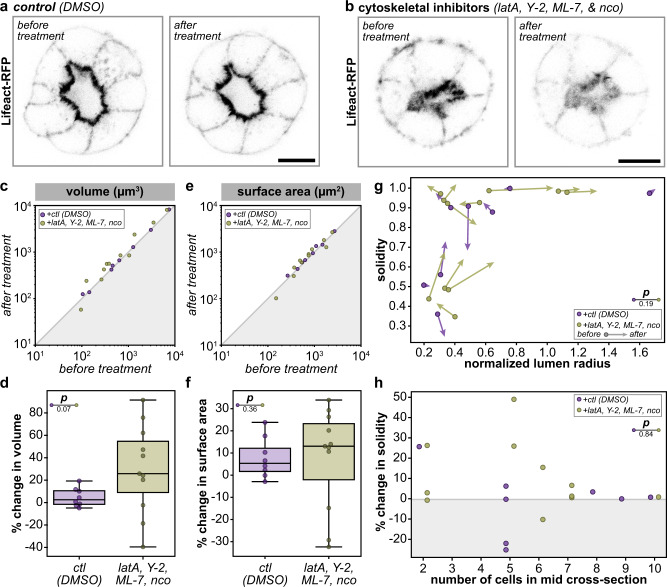
Acute cytoskeletal ablation does not significantly alter lumen shape. **a** Representative single-plane images MDCK spheroids expressing Lifeact-RFP 2 min before (left) and 18 min after (right) addition of DMSO vehicle control. **b** Representative single-plane images MDCK spheroids expressing Lifeact-RFP 2 min before (left) and 18 min after (right) treatment with a cytoskeletal inhibitor cocktail (latrunculin A (latA), Y-27632 (Y-2), ML-7, and nocodazole (nco)). **c** Log–log plot of lumen volume before and after treatment (two-sided rank-sum test DMSO-cytoskeletal inhibitors *p* = 0.13, two-sided Wilcoxon signed rank test *t*_−2 min_ − *t*_+18 min_(DMSO) *p* = 0.263, *t*_−2 min_ − *t*_+18_ min(cytoskeletal inhibitors) *p* = 0.041). **d** Percent change in lumen volume in response to treatment (*p*-value from two-sided rank-sum test on percent change volume). **e** Log–log plot of lumen surface area before and after treatment (two-sided rank-sum test DMSO-cytoskeletal inhibitors *p* = 0.41, two-sided Wilcoxon signed rank test *t*_−2 min_ − *t*_+18 min_(DMSO) *p* = 0.069, *t*_−2 min_ − *t*_+18 min_(cytoskeletal inhibitors) *p* = 0.091). **f** Percent change in lumen surface area in response to treatment (*p*-value from two-sided rank-sum test on percent change surface area). **g** Lumen solidity plotted before (arrow end) and after (arrowhead) treatment with DMSO vehicle control (purple) or cytoskeletal inhibitor cocktail (green) as a function of normalized lumen radius (*p*-value from two-sided 2D Kolmogorov–Smirnov test). **h** Percent change in lumen solidity plotted as a function of numbers of cells in spheroid mid cross-section (*p*-values from two-sided 2D Kolmogorov–Smirnov test). Scale bars are 10 µm. For plots **c**–**h**, *n* = 8 and 11 spheroids for DMSO and cytoskeletal inhibitors conditions, respectively. Source data are provided in the Source Data file.

Cortical tension helps define the shape of cells and could thus be an important modulator of lumen shape. We experimentally decreased cell cortical tension with a cocktail of inhibitors (1 µM latrunculin A, 20 µM ML-7, 20 µM Y-27632, and 50 µM nocodazole) that acutely ablates the actomyosin and microtubule cytoskeletons^27^ (Fig. 3a, b, Supplementary Video 2, and Supplementary Fig. 3a, b). This test also served to determine if intraluminal pressure significantly stabilized lumen shape: if the lumen were under positive pressure, softening the cell cortices would cause the lumen to become rounder, as the luminal pressure would push the apical surfaces outwards. This acute treatment resulted in, on average, a small and statistically insignificant increase in luminal volume (Fig. 3c, d), and no significant change in lumen surface area (Fig. 3e, f). Notably, this treatment did not significantly affect either the volume or sphericity of the whole spheroid, indicating that in this observed time frame cytoskeletal disruption also did not alter the geometry of the spheroid as a whole (Supplementary Fig. 3c, d). Treatment likewise did not alter lumen solidity to a statistically significant degree (Fig. 3 g,h). Thus, the size and overall shape of lumens were both robust to an acute and dramatic perturbation to the cytoskeleton.

### Modulation of apical area alters lumen shape

**Fig. 4:**
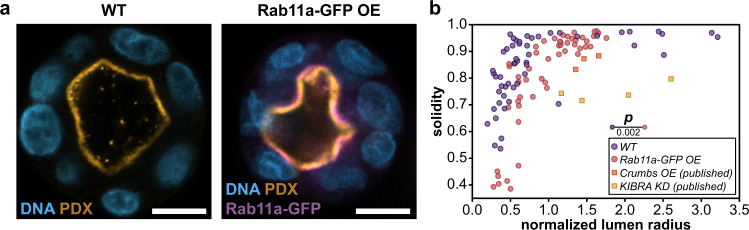
Increasing apical domain size alters lumen shape. **a** Representative images of fixed wildtype (WT, left) and overexpressing Rab11a-GFP (Rab11a-GFP OE, right, purple) MDCK spheroids immunostained for the apical surface protein Podocalyxin (PDX, orange) and DNA (blue). **b** Lumen solidity plotted as a function of normalized lumen radius for wildtype (WT, purple) and apical expansion manipulations (Rab11a-GFP OE red, Crumbs3a overexpression [OE], orange and KIBRA knockdown [KD], yellow; *p*-value from two-sided 2D Kolmogorov–Smirnov test). For plot **b**, *n* = 51 WT, 3 Crumbs3a OE^30^, 4 KIBRA KD^31^, and 51 Rab11a-GFP OE conditions. Scale bars are 10 µm. Source data are provided in the Source Data file.

To test the possibility that the size of the apical domain might influence lumen shape we searched the literature for manipulations that drive the expansion of the apical domain. Trafficking of lipids and membrane proteins is regulated by the Rab family of small GTPases. In MDCK cells, Rab11a was shown to regulate apical membrane trafficking through its interaction with the guanine nucleotide exchange factor Rabin8, and overexpression of Rab11a accelerated lumen formation in MDCK spheroids^1^. To manipulate apical domain size in small spheroids, we generated spheroids of various sizes from Rab11a-GFP overexpressing MDCK cells and measured lumen solidity (Fig. 4a). Rab11a-GFP spheroids generated a significantly larger apical domain and a significantly decreased solidity, compared to wildtype cells with the same number of cells (Fig. 4b, see below). Changes in lumen cross-sectional area were modest, and not statistically significant (Supplementary Fig. 4a).

Apical membrane identity is also determined by the Crumbs and Par complexes^28,29^. Crumbs3a, a Crumbs homolog, is critical for establishment of apical domains and consequently lumen formation in MDCK spheroids^30^. Overexpression of Crumbs3a resulted in apical membrane expansion into the lumen, even when lumens were quite large (estimated radius ~20 µm)^30^. The Par complex is composed of the polarity proteins Par3 and Par6 and the atypical kinase aPKC. Tight regulation of aPKC activity is necessary for proper polarity establishment and maintenance. The membrane-associated protein, KIBRA, an upstream regulator of the YAP/TAZ pathway, can bind to aPKC and inhibit its activity^31^. Consequently, knockdown of KIBRA also results in expansion of the apical domain into the lumen^31^. We measured the shapes of published examples of lumens produced by MDCK cells overexpressing Crumbs3a^30^ or with KIBRA knockdown^31^. In published examples, these manipulations led to a marked decrease in lumen solidity, even at large lumen sizes (Fig. 4b). Thus, regulation of apical domain size could strongly influence lumen shape and size.

### A minimal model of preferred apical domain size can explain features of lumen geometry

The data above suggested that apical domain size, rather than pressure or cortical tension, might play a dominant role determining lumen shape in our system. We sought to develop a physical model that could account for our observations. Although such mathematical models cannot be proven to be correct, they are a useful means of testing the underlying biological model against available data, and of making predictions to guide future experiments.

**Fig. 5:**
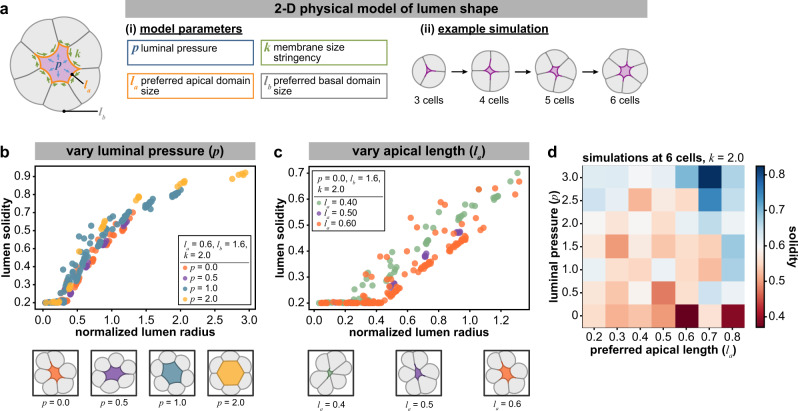
A two-dimensional physical model of lumen shape. **a** Schematic depicting the parameters of a vertex-based model describing lumen shape. **b**, **c** Plots of solidity as a function of normalized lumen radius from simulations of a 2D vertex-based model, keeping preferred apical length and *k* fixed (*l*_a_ = 0.6, *k* = 2.0) while varying luminal pressure (*p* = 0.0–2.0) (**b**), and keeping luminal pressure and cell cortical tension fixed (*p* = 0.0, *k* = 2.0) while varying preferred apical length (*l*_a_ = 0.4–0.6) (**c**). Below plots, representative outputs of simulations as indicated grown to six cells. **d** Heatmap of mean solidity for outputs of simulations while varying luminal pressure and preferred apical length. Source data are provided in the Source Data file.

We adapted a vertex-based model of tissue mechanics to construct a simple two-dimensional model of a growing spheroid^32,33^. This model incorporates intraluminal pressure (*p*), preferred apical and basal domain sizes (*l*_a_, *l*_b_), and a parameter quantifying the stringency with which cells regulate the size of a given membrane domain (*k*) (Fig. 5a). Note that *k* is not synonymous with cortical tension: rather, *k* likely reflects multiple processes that combine to maintain homeostatic control over the sizes of the apical and basal domains. Accordingly, a large value of *k* reflects stringent regulation of membrane domain size, while a low value of *k* reflects less stringent regulation.

Spheroids were simulated as they grew from 3 to 10 cells in size for each set of parameters. Within this model, we systematically varied *p* and *l*_a_, and quantified lumen shape using solidity as a metric. As expected, increasing *p* led to increases in solidity at a given cell number and relative lumen size (Fig. 5b and Supplementary Fig. 5a). Increasing *l*_a_ (preferred apical domain size) led to decreased solidity at low luminal pressures (Fig. 5c and Supplementary Fig. 5b), in agreement with our experiments with Rab11a-GFP cells and our quantification of literature data (Fig. 4b). We note that at higher pressures, the model recapitulates the predictions of a pressure-dependent stabilization of lumen shape, suggesting that the interplay of intraluminal pressure and apical domain regulation may dictate whether a given lumen shape is determined by a pressure-dependent or pressure-independent mechanism (Fig. 5d).

Importantly, lumens tended to have higher solidity with cell number and increasing size even if luminal pressure was zero (Supplementary Fig. 5a, orange), an outcome in line with experimental observation (Fig. 2g). Further, increasing *p* modestly affected lumen cross-sectional area and solidity when the simulations had fewer than five cells; however, as the number of cells increased, a large positive pressure resulted in larger and more regular-shaped lumens (Supplementary Fig. 5a, yellow). These trends agree with the experimentally observed increase in lumen area observed upon treatment with ddAVP (Fig. 2b).

**Fig. 6:**
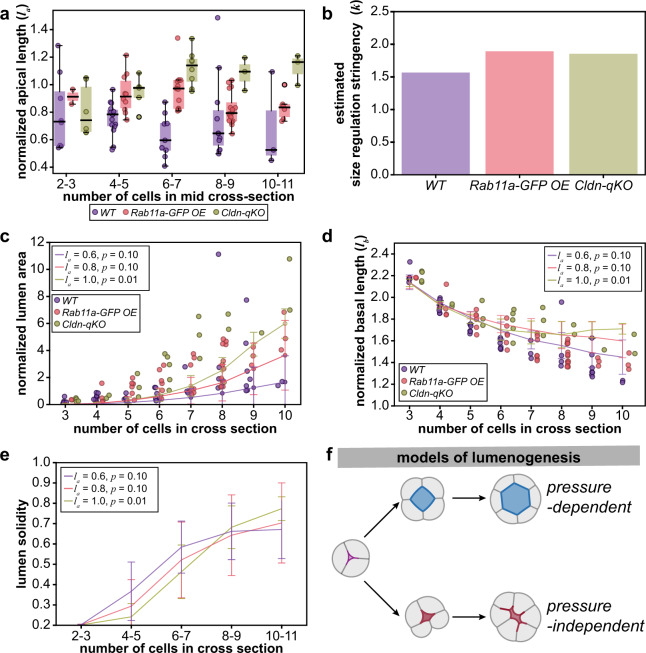
Comparison of model to experimental data. **a**, **b** Estimates were obtained from data for the parameters normalized apical length (*l*_*a*_) (**a**) and size regulation stringency (*k*) (**b**) as described in “Methods” section. The parameters *p* and *l*_b_ were free parameters and tuned to agree with data, with the constraint that *p* for the Cldn-qKO condition was ten-fold smaller than for the other two conditions. **c**, **d** Normalized lumen cross-sectional area (**c**) and normalized mean basal length (**d**) and as a function of size for WT, Rab11a-GFP OE, and Cldn-qKO spheroids. Model predictions (lines) show good quantitative agreement with experimental data (points). Welch’s two-sided *t*-test with Coarsened exact matching (CEM) for cross-sectional lumen area for WT vs. Rab11a-GFP OE *p* = 0.7, WT vs. Cldn-qKO *p* = 5 × 10^−4^. Welch’s two-sided *t*-test with CEM for basal length for WT vs. Rab11a-GFP OE *p* = 0.9, WT vs. Cldn-qKO *p* = 9 × 10^−6^. Welch’s two-sided *t*-test with CEM for simulation cross-sectional area with *l*_a_ = 0.6 vs. 0.8, *p* = 0.06; *l*_a_ = 0.6 vs. 1.0, *p* = 8 × 10^−4^. **e** Plot of model predictions of lumen solidity as a function of number of cells. Welch’s two-sided *t*-test with CEM for simulation cross-sectional area with *l*_a_ = 0.6 vs. 0.8, *p* = 0.50; *l*_a_ = 0.6 vs. 1.0, *p* = 0.38. **f** Schematic depicting pressure-dependent and pressure-independent lumenogenesis. For plots **a**, **c**, and **d** *n* = 51 WT, 51 Rab11a-GFP OE, and 25 Cldn-qKO conditions. Error bars on lines indicate standard deviation for simulation results, center of error bars on lines indicate mean of simulation results, for *n* = 25 simulations for plots **c**, **d**, and **e**. Source data are provided in the Source Data file.

To more directly compare the model predictions with experimental data, we first established a baseline set of model parameters to approximate wildtype data. Among the four free parameters of the model, the preferred apical area *l*_a_ and the parameter *k* could be directly approximated from experimental data (Fig. 6a, b, respectively). We then chose *l*_b_ and *p* to agree with the wildtype data in terms of lumen and total spheroid size. This choice of parameters predicts lumen and total spheroid size for wildtype cells (Fig. 6c, d). We then varied model parameters in accordance with the experimental perturbations to *p* and *l*_a_. As shown in Fig. 4c, both the Cldn-qKO and the Rab11a-GFP cells exhibited an increased *l*_a_. The origin of the increase in apical domain size for the Cldn-qKO cells is not clear. Increasing *l*_a_ from 0.6 to 0.8 as measured in Rab11a-GFP cells modestly decreased solidity and increased relative lumen size (Supplementary Fig. 4a), consistent with experimental data (Fig. 4b). For Cldn-qKO cells, increasing *l*_a_ to 1.0 (Fig. 6a) and decreasing *p* by ten-fold, as expected from measurements of epithelial permeability^26^, had a modest impact on solidity (Fig. 6e). However, the basal domain size and lumen cross-sectional area both increased (Fig. 6c, d), in agreement with measurement and in contrast to a pressure-only model.

While the model can account for the lumen shape in our and other systems, it is incomplete in several ways. First, it models a two-dimensional cross-section of a three-dimensional object. Second, the model assumes the physical properties of the cell’s apical and basolateral surfaces are identical, and that all cells within spheroid have identical sizes and physical properties. Third, it does not provide details as to the mechanism by which apical or basal domain size might be regulated; the two are likely interrelated and jointly influenced by cell–cell adhesion and polarity^5,8,28^. Relatedly, it is possible that lateral domain length might be regulated as well. Finally, the model treats pressure, stringency of membrane area regulation, and preferred apical and basal areas as independent effects, when in reality they may be coordinately regulated. Nonetheless, the model predictions qualitatively agree with our data in ways that pressure-only models cannot. This minimal model is sufficient to capture the trends in our data, and others, regarding lumen shape and size, most notably the increase in lumen size upon knockout of multiple claudins^10,12,13,16^.

## Discussion

Our findings highlight a pressure-independent method for stabilizing lumen shapes that, to our knowledge, has been largely overlooked despite its probable prevalence^20,34–37^. Current evidence demonstrates that hydrostatic pressure plays a central role in the growth and stabilization of lumens in some circumstances, most notably the mammalian blastocyst (Fig. 6f, top). However, we and others find that the Cldn-qKO cells could efficiently form lumens despite the disruption to tight junctions and to epithelial permeability^26^. Instead, our data indicate that lumens can expand via a distinct, pressure-independent pathway in which lumen growth occurs by maintaining a roughly constant amount of apical membrane per cell, with sufficient fluid transport to allow the lumen to gradually increase in volume while avoiding large positive or negative pressures (Fig. 6f, bottom). The advantages of pressure-independent lumen growth remain to be firmly established; however, we note two salient possibilities. First, while pressure-driven growth exposes tissues to pressure-driven rupture^10,13^, a pressure-independent mechanism does not. Second, a back-of-the-envelope calculation (Supplementary Methods) suggests that, for small lumens, the free-energy cost of pressure-driven expansion is greater than that of expansion at low pressure via vesicle fusion.

We developed a physical model that can account for our data as well as pressure-dependent and pressure-independent lumenogenesis in a wide variety of model systems. In this model, the creation of a non-stick apical membrane, which occurs via directed vesicular trafficking^8,28,38^, is itself sufficient to define the contours of the lumen. Lumen shape is however dictated by the balance of preferred apical area and pressure, with high pressure, stringent membrane size regulation, and small apical domains yielding regular shapes. This model can account for the observation of the large variety of both regular and irregular lumen shapes that have been described in different model systems. For example, a high intraluminal pressure can account for round lumen shape in systems such as the mouse blastocyst, while a low pressure and larger preferred apical domain size can account for the irregular lumens in the Rab11a-overexpressing MDCK spheroids. Pressure-dependent and pressure-independent mechanisms for dictating lumen shape thus coexist and fulfill complementary functions in driving embryonic growth and tissue morphogenesis.

Previous work demonstrates that the establishment of apico-basal polarity and the earliest stages in lumen formation are tightly coupled at a molecular level^1,5,8,28^. Our work builds on these observations and highlights the deep connections between the establishment of a distinct, lumen-facing apical membrane and the physical mechanisms that stabilize small lumens. The creation of hollow lumens is likely to be an evolutionarily ancient innovation that was key to the construction of multicellular tissues^39–42^. Evolutionary data indicate the molecular components required for the establishment of a defined apical domain are likewise ancient, and appeared simultaneously with the advent of multicellular animals^43^. We speculate that the evolutionary origins of apico-basal polarization and of lumenogenesis may be inextricably linked, and that their joint appearance constituted a key evolutionary innovation enabling the construction of animal life.

## Methods

### Cells culture and generation of cell lines

MDCK II (Sigma Cat. #00062107) cells were cultured at 37 °C and 5% CO_2_ in DMEM (Thermo Fisher Cat. #11885076) supplemented with 10% fetal bovine serum (FBS, Corning) and 1% penicillin-streptomycin (ThermoFisher). Live-cell confocal and brightfield microscopy experiments were performed in Leibovitz’s L15 media (L15, ThermoFisher) supplemented with 10% FBS (Corning) and 1% penicillin-streptomycin. Live-cell lattice light sheet microscopy was performed in L15 media supplemented with 1% FBS and Insulin-Transferrin-Selenium (Invitrogen). MDCK cells constitutively expressing Lifeact-RFP were generated to visualize the actin cytoskeleton. Briefly, cells were transfected with a plasmid containing the PiggyBac transposon system and the Lifeact-RFP sequence (DNA2.0), cells were selected for plasmid integration with geneticin (G418, ThermoFisher). MDCK Claudin-quintuple knockout (Cldn-qKO) cells were a generous gift from Tetsuhisa Otani and Mikio Furuse (National Institute for Physiological Science, Japan)^26^. MDCK Rab11a-GFP cells were a generous gift from Keith Mostov (UCSF).

### Generating MDCK spheroids

MDCK spheroids were generated as previously described in refs. ^44,45^. Briefly, 75 µL of cell suspension containing ~10^4^ cells were mixed with 150 µL Matrigel GFR (Corning CB-40230). Twenty-five microliter drops of cell-Matrigel suspensions were seeded into each well of an 8-well chambered coverglass (Nunc, No. 1.5) and incubated at 37 °C for 30 min before adding media to cells. Media was changed every other day. At least 2 h prior to live-imaging experiments, media was changed to L15 with supplements (as indicated above).

### Pharmacological inhibition

To disrupt ion and fluid pumping, spheroids were treated with ouabain, a Na^+^/K^+^ ATPase inhibitor (Sigma) or 1-deamino-8-d-arginine vasopressin (ddAVP, Sigma), a vasopressin receptor agonist. Each was dissolved in distilled water at 1000× final concentration and diluted in cell culture media immediately before treatment for 4–24 h before DIC imaging. Ouabain was used at a final concentration of 333 µM while ddAVP was used at a final concentration of 10 µM. For cytoskeletal inhibition (and controls) experiments, data were collected on at least five separate days from distinct samples. For fluid pumping inhibition experiments, data were collected on two separate days from distinct samples.

To perturb the actomyosin and microtubule cytoskeletons, cells were treated with a cytoskeletal inhibitor cocktail composed of latrunculin A (Sigma, 1 µM), Rho-kinase inhibitor Y-27632 (STEMCELL Technologies, 20 µM), MLCK inhibitor ML-7 (Enzo, 20 µM), and nocodazole (Sigma, 50 µM). The cytoskeletal inhibitor cocktail was made at 5× final concentration in L15 media and 100 µL were added to imaging well with 400 µL of L15.

### Cell immunofluorescence

MDCK cells were fixed with 4% paraformaldehyde in PBS for 15 min at room temperature. Samples were blocked and permeabilized in 0.1% Triton, 1% bovine serum albumin (BSA, Sigma) in PBS for 1 h at room temperature. Cells were incubated in primary antibody solution in 0.1% Triton, 1% BSA in PBS overnight at 4 °C, and in secondary antibody solution in 0.1% Triton, 1% BSA in PBS for 2 h at room temperature. To identify nuclei, Hoechst solution (Hoechst 34580, Invitrogen) was added at 1:1000 dilution to secondary antibody solution. Antibodies and corresponding concentrations used in this investigation are listed in Table S1. Images were acquired at room temperature (~22 °C) using an inverted Zeiss LSM 780 confocal microscope with a 40×/1.3 NA C-Apo water objective, 405 nm diode laser, 561 nm diode laser, 633 nm HeNe laser, and a pinhole setting between 1 and 2 Airy Units. All images were acquired using Zen Black software (Carl Zeiss).

### Lattice light sheet microscopy

We used a custom-built lattice light sheet microscope (LLSM)^46^ to image MDCK spheroids. Spheroids were grown in 3 μL droplets of Matrigel without Phenol red (Corning) seeded on top of a 5 mm round cover glass (Warner Instruments). The samples were incubated for 12–36 h at 37 °C in 25 mm tissue culture plates. Before experiments, samples were transferred to LLSM imaging medium (L15 media supplemented with 1% FBS and Insulin-Transferrin-Selenium Invitrogen) for 12–16 h. Data were collected on two separate days from distinct samples.

Samples were illuminated by a 561 nm diode laser (0.5 W, Coherent) using an excitation objective (Special Optics, 0.65 NA with a working distance of 3.74 mm) at 2% AOTF transmittance and laser power of 100 mW. Order transfer functions were obtained empirically by acquiring point-spread functions using 200 nm TetraSpeck beads adhered freshly to 5 mm glass coverslips (Invitrogen T7280) for each wavelength and for each day of experiments.

To achieve structured illumination, a square lattice was displayed on a spatial light modulator. This lattice was generated by an interference pattern of 59 Bessel beams separated by 1.67 µm and cropped to 0.22 with a 0.325 inner NA and 0.40 outer NA. The lattice light sheet was dithered 25 µm to obtain homogeneous illumination with 5% flyback time. Fluorescent signal was collected by a Nikon detection objective (CFI Apo LWD 25XW, 1.1 NA, 2 mm working distance), coupled with a 500 mm focal length tube lens (Thorlabs), a Semrock filter (BL02-561R-25) and sCMOS cameras (Hamamatsu Orca Flash 4.0 v2) with a 103 nm/pixel magnification.

Z-Stacks were acquired by moving the lattice light sheet and the detection objective synchronously, using a galvo mirror coupled at the back focal plane of the illumination objective and a piezomotor, respectively. The slices of the stacks were taken with an interval of 100 nm through ranges of 30–35 μm at 100 ms camera exposure with 1–5 s intervals between z-stacks.

Raw data was flash corrected^47^ and deconvolved using an iterative Richardson-Lucy algorithm (Chen et al.^46^) run on two graphics processing units (NVIDIA, GeForce GTX TITAN 4 Gb RAM). Flash calibration, flash correction, channel registration, order transfer function calculation and image deconvolution were done using the LLSpy open software^48^. Visualization of the images and volume inspection were done using Spimagine (https://github.com/maweigert/spimagine) and ClearVolume^49^.

### Confocal microscopy

All live confocal images were acquired at 37 °C using an inverted Zeiss LSM 780 confocal microscope with a 40×/1.3 NA C-Apo water objective, 561 nm diode laser, and a pinhole setting between 1 and 2 Airy Units. All images were acquired using Zen Black software (Carl Zeiss).

### Differential interference contrast (DIC) microscopy

DIC microscopy was performed at 37 °C using a Nikon Ti-E inverted microscope with a 20×/0.5 NA Plan Fluor CFI objective and N2 DIC prisms. Image acquisition was controlled using Micro-Manager software^50^.

### Image analysis

A Fiji plugin was used to correct 3D drift in all live-cell confocal images^51,52^.

#### Cell number

The number of cells in each spheroid were manually and independently determined by CGV and VTV using the Lifeact-RFP signal to determine cell–cell boundaries.

#### Segmentation and surface shape calculations

Custom-developed Python code was used to detect and segment the lumens (i.e., apical surface) and basal surfaces spheroids. Briefly, lumen and basal surface boundaries were detected in each slice from segmented images using OpenCV. The boundary was parameterized by contour length. *X*-coordinates and *Y*-coordinates of each boundary were fit to Fourier series of varying order up to 15^53^. A Bayesian Information Criterion was used to select the Fourier order to minimize overfitting^54^. The smoothed boundary was used for calculations of local curvature, volume, and surface area. From these calculations sphericity (ψ) was computed as in Eq. 2. Estimated lumen radius was calculated from the lumen volume as:

4 $$r={\left(\frac{3V}{4\pi }\right)}^{\frac{1}{3}},$$

where *V* is lumen volume.

#### Mean curvature

For each voxel on a surface, the smoothed contours in *XY* and *YZ* correspond to 1-dimensional curves on the surface, which are orthogonal at that voxel. The curvatures of these curves were computed using the Fourier representation. From these two curves, the surface mean curvature was estimated as follows: the surface normal vector was estimated as the cross product of the unit tangent vectors to each of these orthogonal cross-sectional curves. Because these two cross-sections are orthogonal, this is an appropriate approximation.

Let a cross-section contour be given by $\underline{\gamma }$ (*s*). The Frenet–Serret formula gives $\frac{d{{\boldsymbol{T}}}}{ds}=k{{{{{\boldsymbol{N}}}}}}$, where **T** and **N** are the unit tangent and normal vectors of the contour, respectively. The curvature can be related to the geodesic (${k}_{{{{{{\rm{g}}}}}}}$) and normal (${k}_{{{{{{\rm{n}}}}}}}$) curvatures by the formula $\frac{d{{\boldsymbol{T}}}}{ds}\,=\,{k}_{{{{{{\rm{g}}}}}}}{{{{{\boldsymbol{t}}}}}}+{k}_{{{{{{\rm{n}}}}}}}{{{{{\boldsymbol{u}}}}}}$, where ${{{{{\boldsymbol{u}}}}}}$ is the surface unit normal vectors, and ${{{{{\mathbf{t}}}}}}={{{{{\mathbf{u}}}}}}\times {{{{{\boldsymbol{T}}}}}}$, which also follows from the Frenet–Serret formula^55^. The normal curvatures of each cross-sectional curve were thus computed from the estimated surface normal vector and the Frenet–Serret normal vector of that curve as ${k}_{{{{{{\rm{n}}}}}}}\,=\,k\vec{N}\cdot \vec{u}$. The mean curvature was then calculated as the mean of the two orthogonal normal curvatures.

#### Determination of fraction concave surface

Each segmented surface (lumen or basal surface) has a distribution of mean curvatures. To calculate the fraction concave surface area, we determined what fraction of each surface had negative (concave) mean curvatures.

#### Determination of mean luminal area per cell

Each segmented lumen surface was divided by the number of cells in the spheroid.

#### DIC image analysis

For each spheroid imaged in DIC, the apical (lumen) and basal surfaces were traced manually in Fiji^51^ using the polygon selection tool. For apical (*a*) and basal (*b*) surfaces, the enclosed area (*A*) and perimeter (*P*) were measured automatically in Fiji. Mean cell thickness was computed as:

5 $$\frac{2({A}_{{{{{{\rm{b}}}}}}}-{A}_{{{{{{\rm{a}}}}}}})}{{P}_{{{{{{\rm{b}}}}}}}+{P}_{{{{{{\rm{a}}}}}}}}.$$

#### Determination of solidity

Solidity metric was calculated using Fiji plugin to measure the calculate and determine the area of the convex hull (*A*_c_) of the lumen shape. Solidity was computed by:

6 $$\frac{A}{{A}_{{{{{{\rm{c}}}}}}}}$$

A solidity of 1 describes a completely convex shape, such as an ellipse or a regular polygon, while lower values reflect varying degrees of surface irregularity. For 3D confocal datasets, the middle XY-plane of lumen was manually chosen for solidity analysis.

#### Determination of normalized lumen radius

For each spheroid, the lumen and spheroid radius (*r*) were determined calculated from the lumen area and spheroid area (*A*), respectively, as follows:

7 $$r=\sqrt{\frac{A}{\pi }}$$

The normalized lumen radius (*r*_norm*.*_) was calculated from these estimated radii as follows:

8 $${r}_{{{{{{{\rm{norm}}}}}}}.}=\frac{{r}_{l}}{{r}_{{{{{{\rm{s}}}}}}}-{r}_{{{{{{\rm{l}}}}}}}},$$

where *r*_l_ is the estimated lumen radius and *r*_s_ is the estimated spheroid radius.

### Vertex-based model

We adapted a vertex-based model of tissue mechanics to construct a simple two-dimensional model of a monolayer of cells surrounding a lumen. This model incorporates only preferred apical and basal areas, cell cortical tension, and intraluminal pressure.

Each cell in an $N$-cell model spheroid was described by four boundaries: a curved basal boundary, two straight lateral boundaries, and a curved apical boundary with preferred length ${l}_{{{{{{\rm{a}}}}}}}$. These boundaries have lengths and enclose a cell area $A$. Thus, the spheroid shape is completely determined by the locations of the apical and basal vertices, and the curvatures of the apical and basal boundaries. An $N$-cell spheroid was thus represented as a $6N$-dimensional vector which records the *x*,*y* positions of each vertex, the apical curvature, and the basal curvature.

For simplicity, we assume that the area of a cell in the spheroid is constant. The effects of size regulation at the basal, lateral, and apical domains are modeled as springs with rest lengths of *l*_b_, 0, and ${l}_{{{{{{\rm{a}}}}}}}$, respectively. Finally, we include an energetic term that favors higher luminal areas, parametrized by a pressure difference between cells and the lumen ${P}_{{{{{{\rm{L}}}}}}}$, which may be set to zero.

Thus, in line with previous descriptions of such vertex-based models^33,56^ the Hamiltonian of the system is given by:

9 $$H=-{P}_{{{{{{\rm{L}}}}}}}{A}_{{{{{{\rm{L}}}}}}}+\mathop{\sum }\limits_{i=1}^{N}{k}_{{{{{{\rm{A}}}}}}}{\left({A}_{{{{{{\rm{i}}}}}}}-{A}_{0}\right)}^{2}+{k}_{{{{{{\rm{l}}}}}}}\left[{\left({l}_{{{{{{\rm{a,i}}}}}}}-{l}_{{{{{{\rm{a}}}}}}}\right)}^{2}+{\left({l}_{{{{{{\rm{b,i}}}}}}}-{l}_{{{{{{\rm{b}}}}}}}\right)}^{2}+{l}_{{{{{{\rm{l,i}}}}}}}^{2}\right]\,$$

This can be nondimensionalized by dividing by the characteristic energy ${k}_{{{{{{\rm{A}}}}}}}{A}_{0}^{2}$, which yields the dimensionless equation:

10 $$\widetilde{H}=-\widetilde{p}\frac{{A}_{L}}{{A}_{0}}+\mathop{\sum }\limits_{i=1}^{N}{\left(\frac{{A}_{i}}{{A}_{0}}-1\right)}^{2}+k\left[{\left(\frac{{l}_{a,i}}{\sqrt{{A}_{0}}}-{\widetilde{l}}_{a}\right)}^{2}+{\left(\frac{{l}_{b,i}}{\sqrt{{A}_{0}}}-{\widetilde{l}}_{b}\right)}^{2}+\frac{{l}_{l,i}^{2}}{{A}_{0}}\right],$$

where we have the following three dimensionless parameters:

11 $$\widetilde{p}\equiv \frac{{P}_{{{{{{\rm{L}}}}}}}}{{k}_{{{{{{\rm{A}}}}}}}{A}_{0}},{{{{{\rm{a}}}}}}\,{{{{{\rm{dimensionless}}}}}}\,{{{{{\rm{pressure}}}}}}$$

12 $$k\equiv \frac{{k}_{{{{{{\rm{l}}}}}}}}{{k}_{{{{{{\rm{A}}}}}}}{A}_{0}\,},{{{{{\rm{a}}}}}}\, {{{{{\rm{dimensionless}}}}}}\, {{{{{\rm{cortical}}}}}}\, {{{{{\rm{tension}}}}}}$$

13 $${\widetilde{l}}_{{{{{{\rm{a}}}}}}}\equiv \frac{{l}_{{{{{{\rm{a}}}}}}}}{\sqrt{{A}_{0}}},{{{{{\rm{a}}}}}}\, {{{{{\rm{dimensionless}}}}}}\, {{{{{\rm{preferred}}}}}}\, {{{{{\rm{apical}}}}}}\, {{{{{\rm{domain}}}}}}\, {{{{{\rm{size}}}}}},{{{{{\rm{and}}}}}}$$

14 $${\widetilde{l}}_{{{{{{\rm{b}}}}}}}\equiv \frac{{l}_{{{{{{\rm{b}}}}}}}}{\sqrt{{A}_{0}}},{{{{{\rm{a}}}}}}\, {{{{{\rm{dimensionless}}}}}}\, {{{{{\rm{preferred}}}}}}\, {{{{{\rm{basal}}}}}}\, {{{{{\rm{domain}}}}}}\, {{{{{\rm{size}}}}}}.$$

Parameters ${A}_{0},\,$*k* and ${\widetilde{l}}_{{{{{{\rm{a}}}}}}}$ were estimated from data as follows: ${{A}_{0}\,{{{{{{\rm{and}}}}}}}\,\widetilde{l}}_{{{{{{\rm{a}}}}}}}$ were directly estimated, respectively, as the average cross-sectional area of a cell; and the average cross-sectional apical length per cell divided by$\,\sqrt{{A}_{0}}$.

The parameter *k* was estimated as follows. Assuming that fluctuations in the system can be approximated by a constant-temperature thermal system at some effective thermal energy scale ${k}_{{{{{{\rm{B}}}}}}}T$, and that cell area and apical length are approximately statistically independent, then the equilibrium distribution of states follows a Boltzmann distribution:

15 $$p({A}_{{{{{{\rm{i}}}}}}},\,{l}_{{{{{{\rm{a,i}}}}}}},\,{l}_{{{{{{\rm{b,i}}}}}}},{l}_{{{{{{\rm{l,i}}}}}}},\,{A}_{{{{{{\rm{L}}}}}}})\propto {e}^{-\frac{H}{kT}}\propto {e}^{-\frac{{k}_{{{{{{\rm{A}}}}}}}{({A}_{{{{{{\rm{i}}}}}}}-{A}_{0})}^{2}}{{k}_{{{{{{\rm{B}}}}}}}T}}{e}^{-\frac{{k}_{{{{{{\rm{l}}}}}}}{({l}_{{{{{{\rm{a,i}}}}}}}-{l}_{a})}^{2}}{{k}_{{{{{{\rm{B}}}}}}}T}}$$

Under these assumptions, the variance in cell areas ${\sigma }_{{{{{{\rm{A}}}}}}}^{2}$is given by $\frac{{k}_{{{{{{\rm{B}}}}}}}T}{2{k}_{{{{{{\rm{A}}}}}}}}$, and, likewise, the variance in apical lengths ${\sigma }_{{la}}^{2}$ is given by $\frac{{k}_{{{{{{\rm{B}}}}}}}T}{{2k}_{{{{{{\rm{l}}}}}}}}$. Thus, the dimensionless parameter *k* was approximated by

16 $$k\approx \frac{{\sigma }_{{{{{{\rm{A}}}}}}}^{2}}{{\sigma }_{{{{{{{\rm{la}}}}}}}}^{2}{A}_{0}}$$

The remaining two parameters $p\,{{{{{\rm{{and}}}}}}}\,{l}_{{{{{{\rm{b}}}}}}}$ were treated as free parameters, chosen to agree with data. Based on published measurements of transepithelial permeability, the value of $p$ for the Cldn-qKO cells was constrained to be tenfold lower than its value in the other conditions.

This nondimensionalized equation was used to simulate a growing spheroid as follows:

1. A 3-cell spheroid was generated with basal vertices evenly spaced around a circle of radius 2; apical vertices chosen independently, uniformly at random from the interior of the unit circle; and apical curvatures chosen uniformly at random from the interval [1, 2].
2. Stochastic gradient descent was used to minimize the energy of this spheroid such that all vertices were within 0.01 dimensionless units of a local minimum.
3. The highest-energy cell was divided by adding an additional apical and basal vertex in the center of the apical and basal boundaries, respectively.
4. The resulting 4-cell spheroid was again energy-minimized using stochastic gradient descent.
5. The process was repeated up to 10-cell spheroids.

Simulations were performed using Python using the NumPy and SciPy libraries.

### Statistics and reproducibility

All experiments were repeated independently at least three times to ensure reproducibility. All representative micrographs are one example of 8–51 biologically independent replicates of the same experiment, of which the remaining micrographs or quantification as a graph are provided in an associated figure panel, or have been described in the main text.

Statistically significant differences between control and drug treatment groups were assessed via a Rank-sum test, as indicated (Figs. 2b, c and 3c, d and Supplementary Figs. 2 and 3). Statistically significant differences between trends of control and drug or wildtype and Cldn-qKO groups were assessed via two-dimensional Kolmogorov–Smirnov test. Data comparing before and after treatment were assessed using the Paired Wilcoxon test (Fig. 3c, d and Supplementary Fig. 3). Rank-sum and Paired Wilcoxon test statistical analyses were performed using the stats module of the SciPy Python package. Two-dimensional Kolmogorov–Smirnov tests was implemented in Python as described in the refs. ^57,58^.

Before statistical comparison, wildtype, Cldn-qKO, and Rab11a-GFP spheroid data were reweighted using exact matching, to account for class imbalance in the number of cells per spheroid cross-section. Statistically significant differences in mean apical length and lumen solidity were then assessed via a weighted Welch’s *t*-test. Exact matching was performed using the Python package cem, an implementation of the original R code for CEM^59^. Weighted Welch’s *t*-test was performed in Python using the stats module of the statsmodels Python package.

### Reporting summary

Further information on research design is available in the Nature Research Reporting Summary linked to this article.

## Supplementary information

Supplementary Information

Description of Additional Supplementary Files

Supplementary Movie 1

Supplementary Movie 2

Reporting Summary

## Data Availability

Data supporting all figures are available within the paper and in the associated Source Data files. Raw microscopy data are available upon request from the corresponding author. Source data are provided with this paper.

## Source data

Source Data

## Code availability

Python analysis procedures are available on https://github.com/AlexDunnLab/lumen-shape
