# Supplementary material for: Physical basis for the determination of lumen shape in a simple epithelium
Source: Nat Commun. 2021 Sep 23;12:5608. doi: 10.1038/s41467-021-25050-3 (PMC8460836; doi:10.1038/s41467-021-25050-3)
Supplement: Supplementary file 1 — Supplementary Information [file 41467_2021_25050_MOESM1_ESM.pdf]

Supplementary Information for:

Physical basis for the determination of lumen shape in a simple epithelium

Claudia G. Vasquez[1, 3], Vipul T. Vachharajani[2, 3], Carlos Garzon-Coral[1], and Alexander R. Dunn[1, 2*]
[1]Department of Chemical Engineering, [2]Biophysics Program
Stanford University, Stanford CA 94305 USA
[3]These authors contributed equally to this work
*Corresponding author. Email: alex.dunn@stanford.edu

This PDF file includes:

Supplementary Figures
- Supplementary Figure 1 2
- Supplementary Figure 2 3
- Supplementary Figure 3 4
- Supplementary Figure 4 5
- Supplementary Figure 5 6

Supplementary Table
- Supplementary Table 1 7

Supplementary Methods 8-9
Supplementary References 9

Supplementary Figures

Supplementary Figure 1

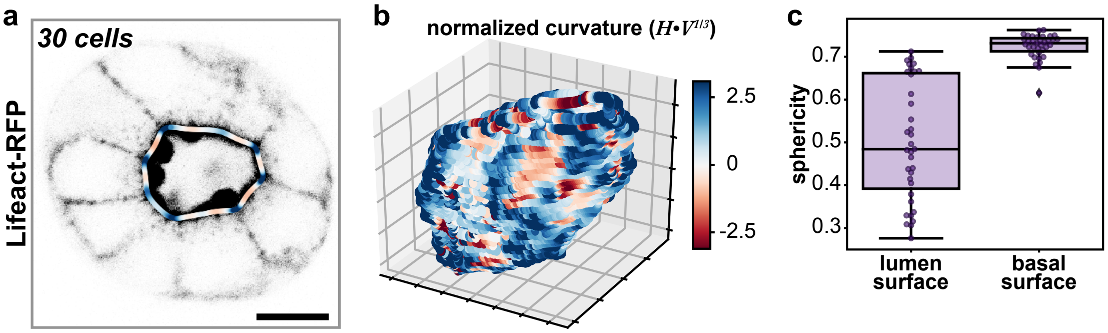

Supplementary Figure 1 | Quantification of lumen surface and basal surface morphology. a, Representative single-plane of MDCK spheroid expressing Lifeact-RFP (gray) with 30 cells surrounding the lumen. The mean lumen curvature is superimposed as a red-blue outline, where red is concave (negative local curvature) and blue is convex (positive local curvature). **b,** 3D contour plot of corresponding lumen surface showing local curvatures, where red is concave (negative local curvature) and blue is convex (positive local curvature). **c,** Sphericity lumen surface (left) and basal surface (right) (*n* = 35 spheroids). Scale bars are 10 µm. Box plot in **c** shows median, quartiles of dataset, and whiskers extending to maximum and minimum of distributions, excluding outliers (indicated with diamonds).

Supplementary Figure 2

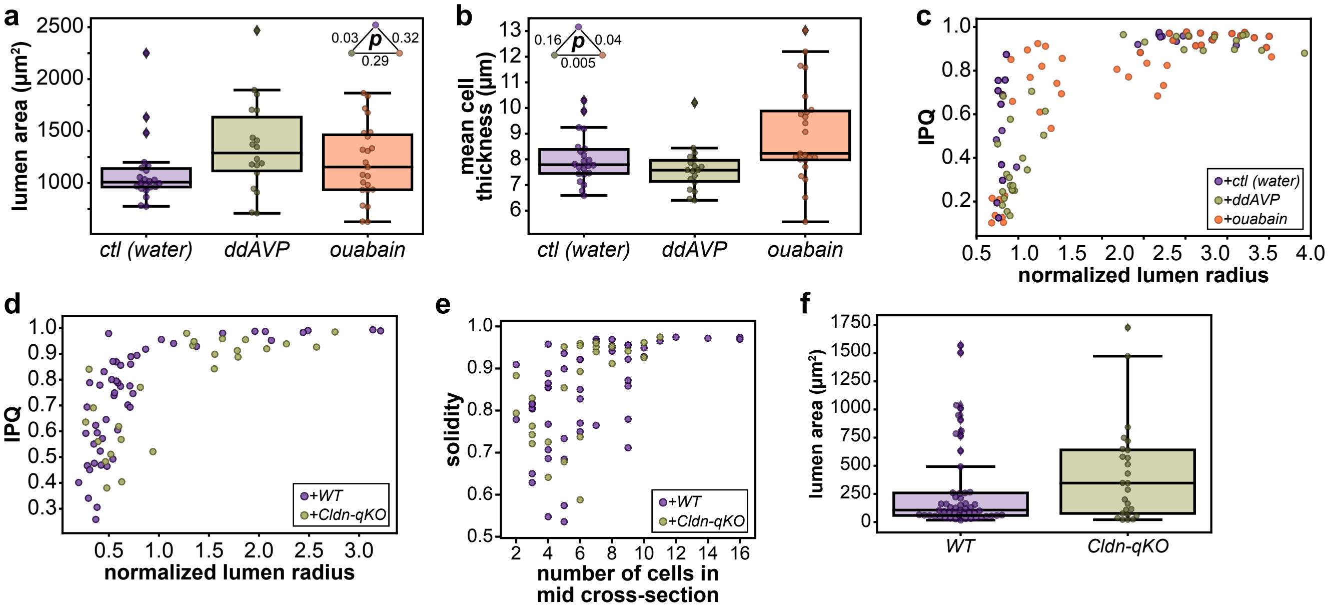

Supplementary Figure 2 | MDCK spheroid response to changes in intraluminal pressure. a-b, Quantification of MDCK spheroids grown in Matrigel for 7 days treated with ddAVP or ouabain for 4 hours. **a,** Quantification of cross-sectional luminal area in control, ddAVP, and ouabain conditions (*p*-values from two-sided rank-sum test). **b,** Quantification of mean cross-sectional cell wall thickness in control, ddAVP, and ouabain conditions (*p*-values from two-sided rank-sum test). **c,** Lumen isoperimetric quotient (IPQ) from MDCK spheroids grown for 3 days and 7 days, treated as indicated with vehicle (purple), ddAVP (green), or ouabain (orange) for 4 hours as a function of normalized lumen radius. **d,** Lumen IPQ plotted as a function of normalized lumen radius for wildtype lumens (WT, purple), Cldn-qKO lumens (green). **e,** Lumen solidity plotted as a function of normalized lumen radius for wildtype lumens (WT, purple), Cldn-qKO lumens (green). **f,** Lumen area plotted as a function of normalized lumen radius for wildtype lumens (WT, purple), Cldn-qKO lumens (green) (two-sided rank-sum test $p = 0.13$). Box plots in **a**, **b**, and **f** show median, quartiles of dataset, and whiskers extending to maximum and minimum of distributions, excluding outliers (indicated with diamonds). For plots **a** and **b**, $n = 15$, 21, 20 spheroids for control, ddAVP, and ouabain conditions, respectively. For plot **c**, $n = 48$, 33, 42 spheroids for control, ddAVP, and ouabain conditions, respectively. For plots **d**-**f**, $n = 51$, 25 spheroids for WT and Cldn-qKO conditions, respectively.

Supplementary Figure 3

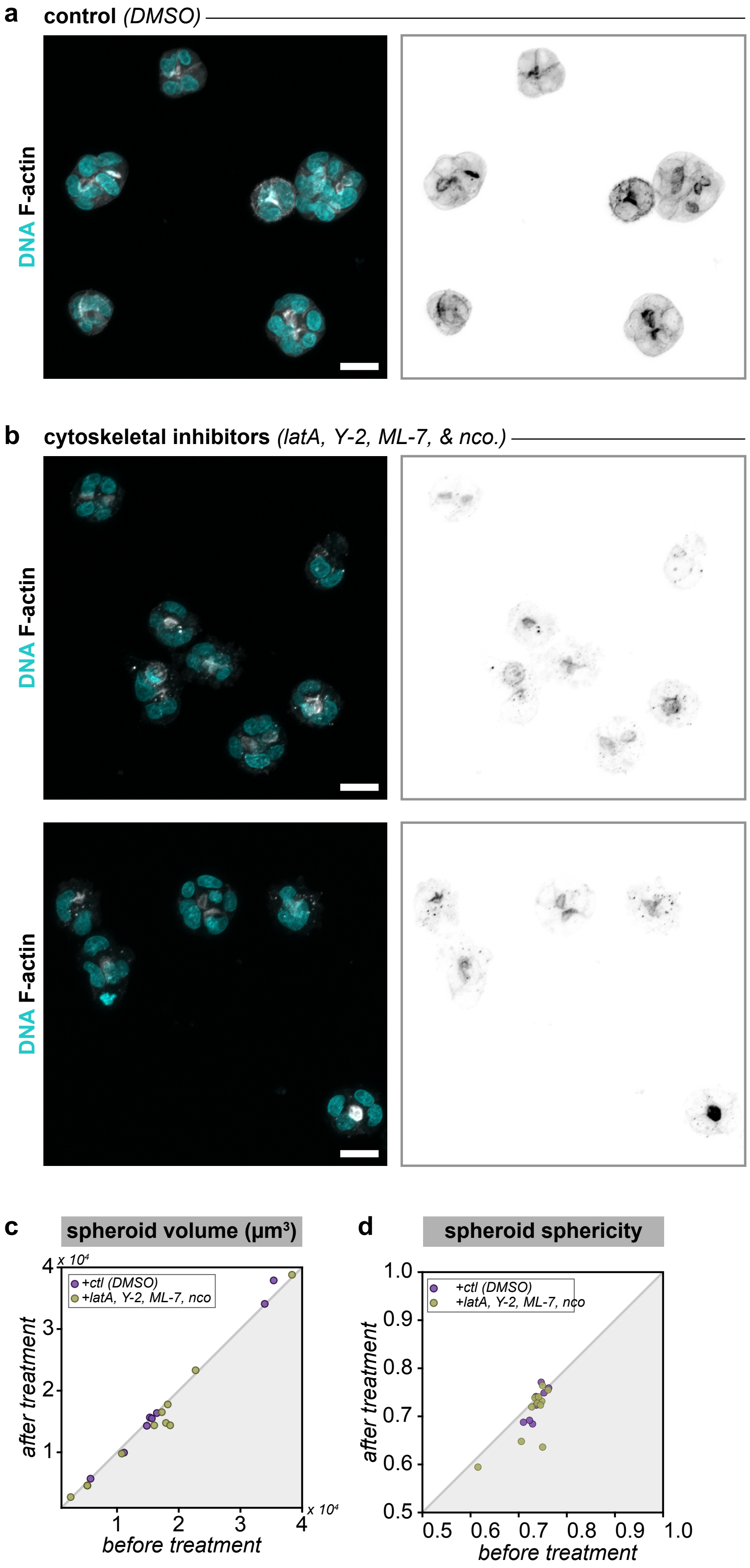

Supplementary Figure 3 | MDCK spheroid response to cytoskeletal ablation. We measured the effects of this cytoskeletal cocktail 18 minutes after addition, as we wanted to find an ideal dose where the cocktail had affected the cortex (by phalloidin staining) without significantly affecting other cellular processes. **a, b,** Representative Z-projections images of MDCK spheroids grown for 2 days and treated for 15 min with DMSO vehicle control **(a)** or cytoskeletal inhibitor cocktail (latrunculin A, Y-27632, ML-7, and nocodazole) **(b)** before fixation and stained for DNA (cyan) and F-actin (phalloidin, gray). Samples stained with same dilutions and images with same intensity scales. **c,** Plots of spheroid volume before and after treatment (two-sided rank-sum test DMSO-cytoskeletal inhibitors $p = 0.13$, two-sided Wilcoxon signed rank test $t_{-2\,min}$-$t_{+18\,min}$(DMSO) $p = 1$, $t_{-2\,min}$-$t_{+18\,min}$(cytoskeletal inhibitors) $p = 0.02$). **d,** Plot of spheroid sphericity before and after treatment (two-sided rank-sum test DMSO-cytoskeletal inhibitors $p = 0.74$, two-sided Wilcoxon signed rank test $t_{-2\,min}$-$t_{+18\,min}$(DMSO) $p = 0.21$, $t_{-2\,min}$-$t_{+18\,min}$(cytoskeletal inhibitors) $p = 0.02$). Scale bars are 20 µm. For plots **c** and **d**, **n** = 8 and 11 spheroids for DMSO and cytoskeletal inhibitors conditions, respectively.

Supplementary Figure 4

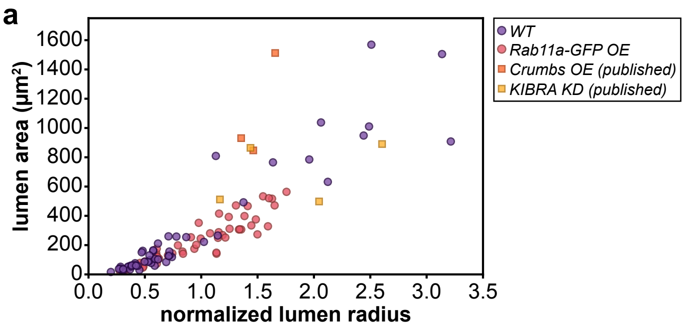

Supplementary Figure 4 | Lumen area does not change in response to apical expansion. a, Plot of lumen area as a function of normalized lumen radius for wildtype (WT, purple) and apical expansion manipulations (Rab11a-GFP OE red, Crumbs3a overexpression [OE], orange and KIBRA knockdown [KD], yellow). For plot, *n* = 51 WT, 3 Crumbs3a OE[30], 4 KIBRA KD[31], and 51 Rab11a-GFP OE conditions.

Supplementary Figure 5

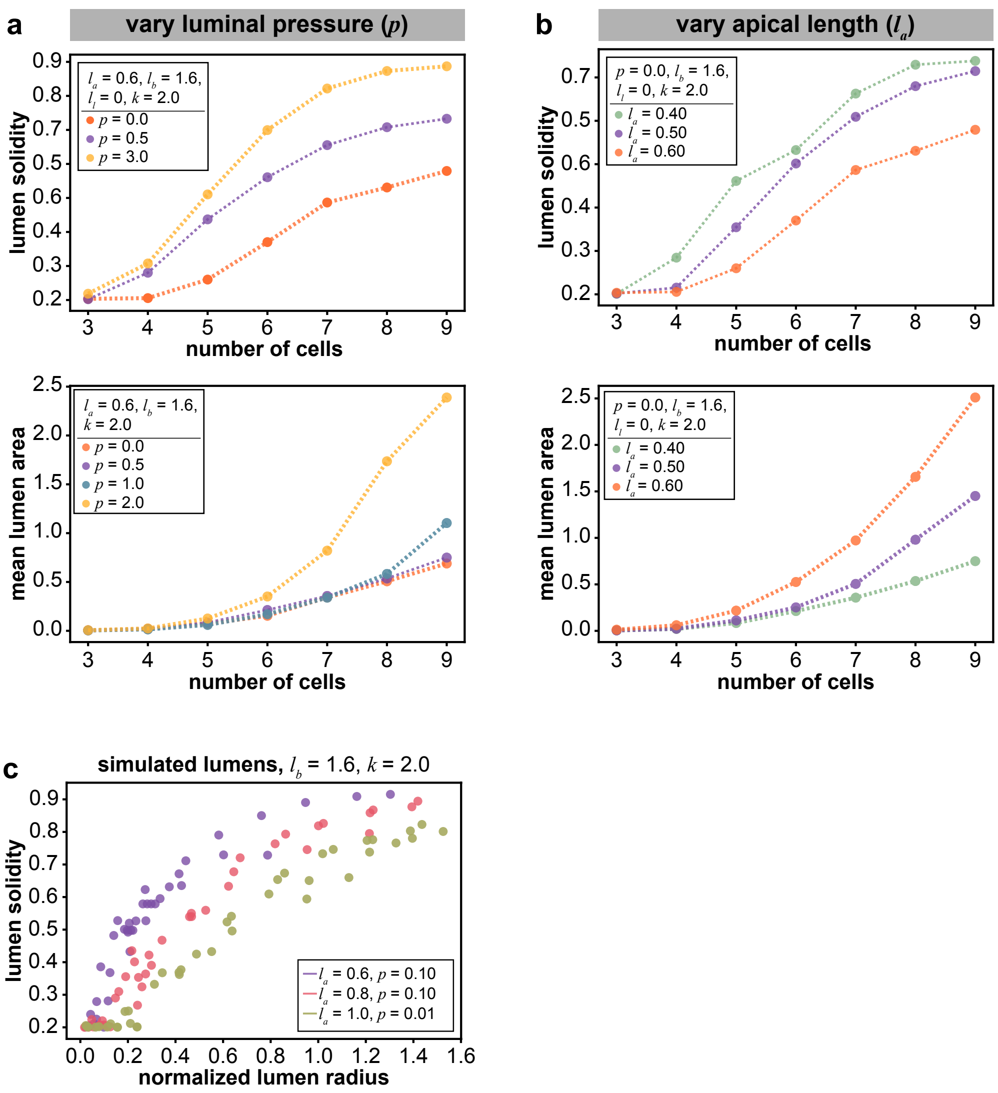

Supplementary Figure 5 | Simulation outputs of two-dimensional physical model of lumen shape. a and **b,** Plots of physical model simulation outputs keeping preferred apical length and size regulation stringency *k* fixed (l_a=0.6, *k*=2.0) while varying luminal pressure (*p*=0.0-3.0) **(a)**, and keeping luminal pressure and *k* fixed (*p*=0.0, *k*=2.0) while varying preferred apical length (l_a=0.4-0.6) **(b)**. (first row) Plots of solidity as a function of number of cells from simulations of 2D vertex-based model. (second row) Plots of mean lumen cross-sectional area as a function of number of cells in a spheroid from simulations of 2D vertex-based model. **c,** Plot of lumen solidity as a function of normalized lumen radius from simulations of a 2D vertex-based model keeping preferred basal length (l_b) and *k* fixed (l_b=1.6, *k*=2.0) while varying luminal pressure (*p*) and apical length (l_a) as indicated.

Supplementary Table

Table S1| Antibodies used and concentrations

Antibody	Dilution	Source
mouse anti-gp135 (PDX)	1:50	Developmental Studies Hybridoma Bank (3F2/D8)
goat anti-mouse IgG Alexa Fluor 555	1:1000	Cell Signaling Technology (Cat. 4413)
goat anti-mouse IgG Alexa Fluor 647	1:1000	Cell Signaling Technology (Cat. 4410)

Supplementary Methods

We will consider the free energy cost of membrane deformation associated with adding a small volume to a lumen via either pumping of isotonic fluid or by fusing small vesicles.

Isotonic fluid pumping

To pump isotonic fluid, mechanical work is done on the spheroid in three ways: first, the resulting fluid may do pressure-volume work against a gradient in hydrostatic pressure. Second, the apical membrane may deform to accommodate more fluid. That is, the work done to pump fluid into the lumen can be expressed as

$$\delta w_p \approx \delta w_{PV} + \delta w_{membrane}$$

The pressure-volume work done against a hydrostatic pressure gradient P is simply $\frac{\delta w_{PV}}{\Delta V} = P$. Hydrostatic pressure gradients in MDCK cell domes have been measured at substantially less than 1kPa[12], for which we have

$$\frac{\delta w_{PV}}{\Delta V} \leq 1kPa = 0.001\frac{kJ}{L}$$

Finally, deformation of the apical membrane is due to bending and area expansion. If the added volume is much smaller than the total lumen volume, then we can neglect bending energy of the membrane. If the lumen is “under-full,” i.e. its volume is less than that of a sphere with the same surface area, then there is no surface area change upon fluid pumping. If the lumen is spherical, then pumping additional fluid requires that the apical membrane expand in area. The work done to stretch the apical membrane is given by

$$\frac{\delta w_{membrane}}{\Delta V} = K_e \frac{\Delta A}{\Delta V}$$

for area expansion modulus K_e. For mammalian cells, this has been measured at 0.1-1 N/m. From geometry, we have for a spherical lumen of radius *r* that $\frac{\Delta A}{\Delta V} \approx \frac{8\pi r \Delta r}{4\pi r^2 \Delta r} = \frac{2}{r}$, and thus for K_e = 0.25

$$\frac{\delta w_{membrane}}{\Delta V} \approx \frac{2K_e}{r} \approx 0.5\frac{kJ \cdot \mu m}{L} \cdot \frac{1}{r}$$

So the work done in pumping isotonic fluid varies as the lumen radius, and is at least

$$\delta w_p \approx \left(0.001\frac{kJ}{L} + \frac{0.5}{r}\frac{kJ \cdot \mu m}{L}\right) \cdot \Delta V$$

For a “typical” vesicle of radius 50-100 nm, the work done in pumping an equivalent volume directly is

$$\delta w_p \approx \left(0.001\frac{kJ}{L} + \frac{0.5}{r}\frac{kJ \cdot \mu m}{L}\right) \cdot 5.2 \cdot 10^{-19} L \approx \frac{(2.6 \cdot 10^{-19})}{r} kJ \cdot \mu m$$

Note that the simplification rests on the assumption that the hydrostatic work term is small, which is reasonable given the low pressures generated by MDCK lumens.

Vesicle fusion

The net mechanical bending work done on the membrane to generate a vesicle[60] is on the order of

$$\delta w_v \approx 8\pi \cdot (250\ k_B T) \approx 6000 \cdot 4.1\, pN \cdot nm = 2.5 \cdot 10^{-20}\ kJ$$

Assuming the membrane with which the vesicle is fusing is not under tension, this is the net work done to fuse one vesicle of fluid.

To compare the marginal work cost of pumping a small amount of fluid into the lumen and fusing a small vesicle, we ask at what radius $\delta w_p < \delta w_v$. This crossover happens when

$$\frac{(2.6 \cdot 10^{-19})}{r} kJ \cdot \mu m < 2.5 \cdot 10^{-20}\ kJ$$

$$r > 10.4\ \mu m$$

This implies that for lumens smaller than about 10 microns in radius, it is preferable to add volume by vesicle trafficking, resulting in a non-spherical lumen. However, when the lumen radius is more than about 10 microns, it becomes less marginally-costly to pump isotonic fluid into a spherical lumen than to make and fuse vesicles, permitting the maintenance of a spherical lumen without performing excessive mechanical work on the membrane.

Supplementary References

60. Phillips, R., Kondev, J. & Theriot, J. *Physical Biology of the Cell* (Garland Science, Taylor & Francis Group, 2008).
