# Supplementary material for: Physical basis for the determination of lumen shape in a simple epithelium
Source: Nat Commun. 2021 Sep 23;12:5608. doi: 10.1038/s41467-021-25050-3 (PMC8460836; doi:10.1038/s41467-021-25050-3)
Supplement: Supplementary file 2 — Description of Additional Supplementary Files [file 41467_2021_25050_MOESM2_ESM.pdf]

Description of Additional Supplementary Files

File Name: Supplementary Movie 1
Description: Representative LLSM movie of 2-celled MDCK spheroid expressing Lifeact-RFP showing non-adherent apical surfaces. Left pane is a XY slice through the lumen and the right plane is a YZ slice of the same spheroid. Scale bar is 5 µm, movie lasts 10 s in real time.

File Name: Supplementary Movie 2
Description: Representative movies of MDCK spheroids expressing Lifeact-RFP treated with DMSO vehicle control (**left**) and cytoskeletal inhibitor cocktail (latrunculin A, Y- 27632, ML-7, and nocodazole, **right**). Scale bar is 10 µm.
