# Supplementary material for: Physical basis for the determination of lumen shape in a simple epithelium
Source: Nat Commun. 2021 Sep 23;12:5608. doi: 10.1038/s41467-021-25050-3 (PMC8460836; doi:10.1038/s41467-021-25050-3)
Supplement: Supplementary file 5 — Reporting Summary [file 41467_2021_25050_MOESM5_ESM.pdf]

nature research

Corresponding author(s): Alexander Dunn

Last updated by author(s): YYYY-MM-DD

Reporting Summary

Nature Research wishes to improve the reproducibility of the work that we publish. This form provides structure for consistency and transparency in reporting. For further information on Nature Research policies, see our Editorial Policies and the Editorial Policy Checklist.

Statistics

For all statistical analyses, confirm that the following items are present in the figure legend, table legend, main text, or Methods section.

n/a	Confirmed	
☐	☒	The exact sample size (*n*) for each experimental group/condition, given as a discrete number and unit of measurement
☐	☒	A statement on whether measurements were taken from distinct samples or whether the same sample was measured repeatedly
☐	☒	The statistical test(s) used AND whether they are one- or two-sided<br>*Only common tests should be described solely by name; describe more complex techniques in the Methods section.*
☒	☐	A description of all covariates tested
☒	☐	A description of any assumptions or corrections, such as tests of normality and adjustment for multiple comparisons
☐	☒	A full description of the statistical parameters including central tendency (e.g. means) or other basic estimates (e.g. regression coefficient) AND variation (e.g. standard deviation) or associated estimates of uncertainty (e.g. confidence intervals)
☐	☒	For null hypothesis testing, the test statistic (e.g. *F*, *t*, *r*) with confidence intervals, effect sizes, degrees of freedom and *P* value noted<br>*Give P values as exact values whenever suitable.*
☒	☐	For Bayesian analysis, information on the choice of priors and Markov chain Monte Carlo settings
☒	☐	For hierarchical and complex designs, identification of the appropriate level for tests and full reporting of outcomes
☒	☐	Estimates of effect sizes (e.g. Cohen's *d*, Pearson's *r*), indicating how they were calculated

Our web collection on statistics for biologists contains articles on many of the points above.

Software and code

Policy information about availability of computer code

Data collection	Confocal microscopy images were acquired using Zeiss Zen Black software (2.0). DIC images were acquired using Micromanager 1.4. LLSM images were acquired with a custom-built LabView (9) software. Mathematical modeling was performed using Python 3.6 (NumPy 1.18.2 and SciPy 1.4.1).
Data analysis	LLSM data were analyzed using LLSPy, Spimagine, and ClearVolume. Confocal images were visualized with ClearVolume and analyzed using FIJI and custom Python 3.7 scripts using open-source packages (scikit-image 0.16.2, NumPy 1.17.2, SciPy 1.3.1). DIC images were analyzed using FIJI. All statistical analyses were performed in Python (SciPy 1.3.1).

For manuscripts utilizing custom algorithms or software that are central to the research but not yet described in published literature, software must be made available to editors and reviewers. We strongly encourage code deposition in a community repository (e.g. GitHub). See the Nature Research guidelines for submitting code & software for further information.

Data

Policy information about availability of data

All manuscripts must include a data availability statement. This statement should provide the following information, where applicable:

- Accession codes, unique identifiers, or web links for publicly available datasets
- A list of figures that have associated raw data
- A description of any restrictions on data availability

Source data for figures are provided with the paper.

Field-specific reporting

Please select the one below that is the best fit for your research. If you are not sure, read the appropriate sections before making your selection.

☒ Life sciences ☐ Behavioural & social sciences ☐ Ecological, evolutionary & environmental sciences

For a reference copy of the document with all sections, see nature.com/documents/nr-reporting-summary-flat.pdf

Life sciences study design

All studies must disclose on these points even when the disclosure is negative.

Sample size	No particular statistical method was used to define sample size. A minimum of n=3 independent experiments were carried out. Sample size was determined based on previous studies in the field (Latorre et al Nature 2018; Bryant et al NCB 2010).
Data exclusions	The only data that were excluded were if image quality was too poor (the structure of interest did not remain in the field of view throughout the whole imaging process)for the automated analysis pipeline.
Replication	For cytoskeletal inhibition experiments, data were collected on at least 5 separate days from distinct samples. For fluid pumping inhibition experiments, data were collected on two separate days from distinct samples. For lattice light-sheet microscopy, data were collected on two separate days from distinct samples. All attempts at replication were successful.
Randomization	In experiments not involving grouping, no randomization was performed. In experiments involving grouping, no particular randomization strategy was implemented.
Blinding	Blinding was not possible because the investigators who conducted the experiments also carried out data analysis (using custom code).

Reporting for specific materials, systems and methods

We require information from authors about some types of materials, experimental systems and methods used in many studies. Here, indicate whether each material, system or method listed is relevant to your study. If you are not sure if a list item applies to your research, read the appropriate section before selecting a response.

Materials & experimental systems

n/a	Involved in the study	
☐	☒	Antibodies
☐	☒	Eukaryotic cell lines
☒	☐	Palaeontology and archaeology
☒	☐	Animals and other organisms
☒	☐	Human research participants
☒	☐	Clinical data
☒	☐	Dual use research of concern

Methods

n/a	Involved in the study	
☒	☐	ChIP-seq
☒	☐	Flow cytometry
☒	☐	MRI-based neuroimaging

Antibodies

Antibodies used	Primary antibody<br>mouse anti-gp135 (PDX) from Developmental Studies Hybridoma Bank (3F2/D8)<br><br>Secondary antibody<br>goat anti-mouse IgG Alexa Fluor 555, Cell Signaling Technology (Cat. 4413), used at 1:1000<br>goat anti-mouse IgG Alexa Fluor 647, Cell Signaling Technology (Cat. 4410), used at 1:1000<br><br>Also stated in "Cell immunofluorescence" section of Methods
Validation	gp-135 (PDX) antibody validated by knockdown in MDCK cells in 10.1083/jcb.200407072

Eukaryotic cell lines

Policy information about cell lines

Cell line source(s)	Madin Darby Canine Kidney (MDCK) II cells were purchased from Sigma (Cat. # 00062107). MDCK Lifeact-RFP were developed in this study using PiggyBac transfeciton of Lifeact-RFp plasmid (see Methods section). MDCK Claudin-quintuple knockout cells were obtained from M. Furuse lab -- published in (Otani et al JCB 2019). Rab11a-GFP cells were obtained from K. Mostov lab -- published in (Bryant et al NCB 2010).

Authentication	Cell lines were authenticated by supplier (Sigma) or providing laboratories.
Mycoplasma contamination	Cell lines tested negative for myocoplasma (by PCR test).
Commonly misidentified lines<br>(See ICLAC register)	No commonly misidentified cells were used.
